# Protective effect of menthol against diethylnitrosamine-induced hepatocellular carcinoma in mice by downregulating *CTNNB1* and *HIF-1α*

**DOI:** 10.22038/ajp.2024.25230

**Published:** 2025

**Authors:** Zahra Mollaei, Masoumeh Asle-Rousta, Golnaz Asaadi Tehrani

**Affiliations:** 1 *Department of Genetics, Zanjan Branch, Islamic Azad University, Zanjan, Iran*; 2 *Department of Physiology, Zanjan Branch, Islamic Azad University, Zanjan, Iran*; 3 *Aerospace and Mechanical Engineering Department, Notre Dame University, Indiana, USA*

**Keywords:** Monoterpenes, Wnt signaling pathway, Angiogenesis, Alpha-fetoprotein, Liver

## Abstract

**Objective:**

This study examined the impact of menthol, a natural monoterpene, on diethylnitrosamine (DEN)-induced molecular and histopathological changes in the livers of male mice.

**Materials and Methods:**

Forty male mice were divided into four groups: Control, Menthol (M), DEN, and DEN-M. The DEN and DEN-M groups received an intraperitoneal injection of DEN (25 mg/kg) at the age of 14 days. The M and DEN-M groups were also given menthol (50 mg/kg, three times a week for six months) via gavage. The expression of genes related to liver carcinoma was analyzed using real-time PCR. Subsequently, the liver tissue was microscopically examined following staining with hematoxylin-eosin.

**Results:**

After one month, menthol reduced the infiltration of inflammatory cells in the liver tissue of mice injected with DEN. It also prevented the increase in the expression of alpha-fetoprotein (*AFP*) (p<0.001), programmed cell death 6 (p<0.05), hypoxia-inducible factor-1 alpha (*HIF-1α*) (p<0.001), and vascular endothelial growth factor (*VEGF*) (p<0.001) in DEN-M animals compared with DEN group. After six months of session, the expression of *AFP* (p<0.05), *HIF-1α* (p<0.05), secreted frizzled-related protein 1 (p<0.001), and catenin beta 1 (p<0.01) was lower in group DEN-M compared with group DEN. Menthol also partially prevented DEN-induced various histopathological changes in the liver after six months of treatment.

**Conclusion:**

We concluded that menthol inhibits Wnt signaling and suppresses the expression of *HIF-1α *and *VEGF* in the liver of DEN-injected mice. It is probably a suitable option for the prevention and treatment of hepatocellular carcinoma.

## Introduction

Hepatocellular carcinoma (HCC) is a type of cancer that is prevalent worldwide and ranks fifth in terms of occurrence and third in terms of cancer-related deaths. About one million people are diagnosed with HCC every year. Chronic liver diseases are known to contribute to the development of HCC (Llovet et al., 2022). One way to study HCC is by inducing tumors in rodents using chemicals, such as diethylnitrosamine (DEN), an organic compound capable of causing liver cancer like other nitrosamines (Schulien and Hasselblatt, 2021). Injecting DEN into two-week-old male mice intraperitoneally is considered one of the most effective methods of inducing HCC, leading to tumor formation after six months (Connor et al., 2018). The liver of mice aged between 7 to 15 days is more susceptible to DEN-induced dealkylating activity, and gender also plays a significant role in tumorigenesis, with estrogens promoting and androgens inhibiting liver tumor formation (Tolba et al., 2015). DEN is metabolized in the liver by cytochrome P450 enzymes, forming a reactive intermediate that produces alkylated metabolites, ultimately leading to DNA methylation. DEN also induces oxidative stress, worsening DNA damage and increasing mutagenesis. Moreover, DEN activates various signaling pathways such as MAPK/activator protein 1 (AP-1), interleukin-6 (IL-6) /glycoprotein 130/Janus kinase/Signal transducers and activators of transcription-3 (STAT-3), Nuclear factor kappa B (NF-κB), rat sarcoma virus (Ras)/rapidly accelerated fibrosarcoma (Raf)/ MAPK/extracellular signalregulated kinase (ERK), Wnt/β-catenin, or phosphatidylinositol 3-kinases (PI3K)/protein kinase B/mammalian target of rapamycin (mTOR) leading to inflammation and subsequently the development of HCC (Schulien and Hasselblatt, 2021). 

Studies have revealed that many drugs used to treat cancer, are derived from natural plant products (Rawat et al., 2018). Menthol [5-methyl-2-(1-methylethyl)cyclohexanol; 2-isopropyl-5-methylcyclohexanol or p-methan-3-ol] is a type of natural monoterpene that is abundantly found in corn mint oil (Kamatou et al., 2013). Menthol is known to possess antioxidant, anti-inflammatory (Rozza et al., 2014), and hepatoprotective (Matouk et al., 2022) properties. Recently, researchers have discovered that menthol also has cytotoxic effects on various cancer cell lines, making it a potential anticancer agent (Singh et al., 2023; Zhao et al., 2023). 

This study investigated the effects of menthol on HCC in mice induced by DEN, using molecular and histological methods.

## Materials and Methods

### Animals and research outline

This study involved forty male mice that were fourteen days old and kept in standard laboratory conditions. The research was approved by the ethics committee of the Islamic Azad University of Zanjan, which followed the ethical principles for working with laboratory animals (code of ethics IR.IAU.Z.REC.1401.017). The mice were divided into four groups, each containing ten mice: 

- Control group (no treatment). 

- Menthol group (M): received menthol (50 mg/kg, three times per week) by gavage for six consecutive months (Santo et al., 2021). 

- Diethylnitrosamine (DEN) group underwent a single intraperitoneal (IP) injection of DEN (25 mg/kg) at fourteen days of age (Tolba et al., 2015; Connor et al., 2018). 

- DEN-M group received menthol treatment for six consecutive months, starting one day after intraperitoneal injection of DEN at fourteen days old. 

**Figure 1:**
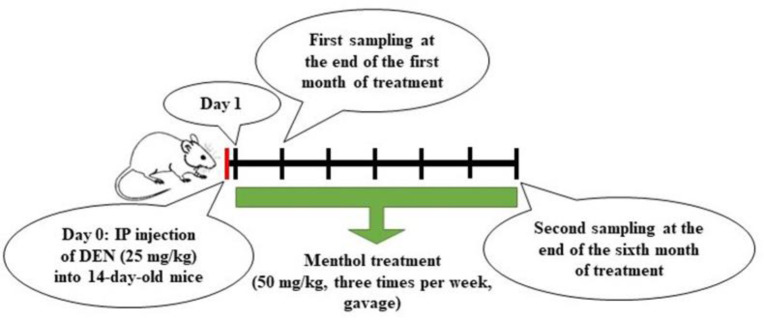
Research Outline

The study lasted six months. At the end of the first and sixth months, five mice from each group were sacrificed by cervical dislocation under ketamine (100 mg/kg, IP)/xylazine (10 mg/kg, IP) anesthesia, and their livers were removed (Figure 1). Liver samples were used for histological examinations, as well as for determining the expression of alpha-fetoprotein (*AFP*), catenin beta 1 (*CTNNB1*), secreted frizzled-related protein-1 (*SFRP1*), hypoxia-inducible factor-1 alpha (*HIF-1α*), vascular endothelial growth factor (*VEGF*), and programmed cell death 6 (*PDCD6*) genes using real-time polymerase chain reaction (PCR).

### Real-time PCR

First, total RNA was extracted using the Parstous RNA extraction kit (Iran). The concentration and quality of extracted RNA were determined by spectrophotometry at 260 nm and by the ratio A260/A280. Then cDNA was produced according to the instructions of the Easy cDNA Synthesis Kit (Parstous, Iran). Real-time PCR was performed with a Rotor-Gene Q (Qiagen, Germany) using qPCRBIO SyGreen Mix Lo-ROX (PCR BIOSYSTEMS, UK). The thermal cycling conditions included one cycle of primary denaturation (95°C for 2 min), 40 cycles of denaturation (95°C for 30 sec), 40 cycles of annealing (52°C for 30 sec), and 40 cycles of extension (72°C for 20 sec). The analysis of melting curves was conducted to verify the single PCR product of each primer. The primers were synthesized by GenFanAvaran (Iran), and their sequences are shown in Table 1. The 2^-ΔΔCT^ method was used to normalize the amplification of each target to its corresponding mRNA levels of glyceraldehyde‐3‐phosphate dehydrogenase (GAPDH) (Livak and Schmittgen, 2001). 

### Histopathological investigations

Tissue samples were taken from the same regions of the animals' livers. The liver samples were fixed with 10% formalin and then, embedded in paraffin. Afterward, 5-micrometer thick slices were prepared and stained with hematoxylin-eosin. Lastly, ten sections of each animal's liver were randomly examined under a microscope. The degree of tissue inflammation was determined semi-quantitatively. This was done using a ranking system where inflammation was categorized as follows: 

(-) no immune cell infiltration, (±) immune cell infiltration in less than 5% of fields, (+) immune cell infiltration in less than 20% of fields, (++) immune cell infiltration in 20 to 60% of fields, and (+++) immune cell infiltration in over 60% of fields (Abdollahi et al., 2024). 

### Statistical analyses

The data are presented as means ± the standard error of the mean (SEM) for each group. To detect any differences between experimental groups, a one-way analysis of variance was conducted. The variance between groups was determined using the LSD and Tukey *post-hoc* test. A statistical significance index of p<0.05 was considered.

## Results

### The effect of menthol on the expression of AFP, PDCD6, SFRP1, CTNNB1, HIF-1 and VEGF in the liver of mice received DEN

**Figure 2:**
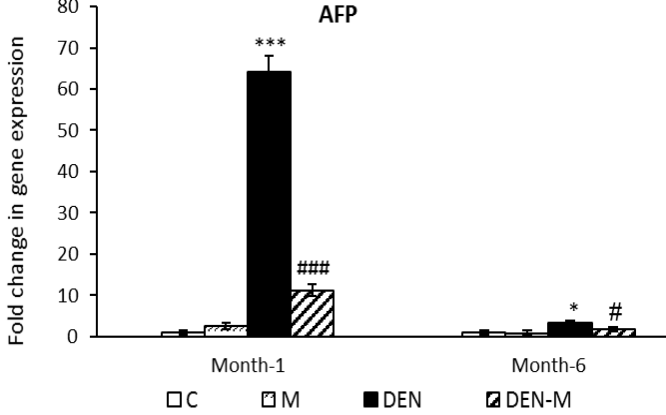
Effect of menthol on mRNA expression of AFP in the liver of mice that received DEN. Results are shown as means ± SEM. Each group contained 5 mice. *p<0.05 and ***p<0.001 vs. the control group and #p<0.05 and ###p<0.001 vs. the group DEN.

After the first month, mice injected with DEN showed a significant increase of 64.14 times in *AFP* mRNA level in their liver compared to the control group (p<0.001). However, menthol treatment prevented this increase in the DEN-M group, resulting in *AFP* expression only reaching 11.20 times that of the control group, which was significantly lower than the DEN group (p<0.001). By the end of the sixth month, the expression of *AFP* in the DEN group was 3.20 times that of the control group (p<0.05), but the menthol treatment greatly prevented its increase. In the DEN-M group, *AFP* expression was only 1.74 times the control group at the end of the sixth month (p<0.05) (Figure 2). There was no significant difference in *AFP* expression between the M group and the control group in both sampling.

**Figure 3:**
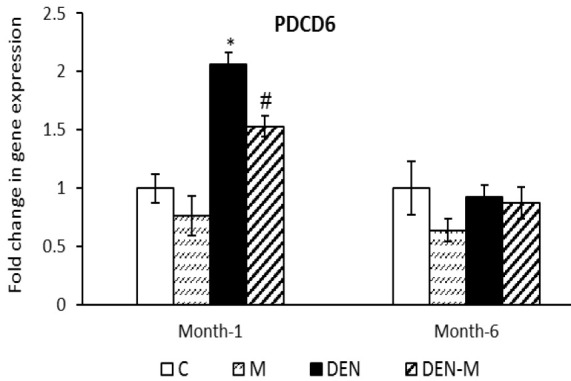
Effect of menthol on mRNA expression of PDCD6 in the liver of mice that received DEN. Results are shown as means ± SEM. Each group contained 5 mice. *p<0.05 vs. the control group and #p<0.05 vs. the group DEN.

At the end of the first month, the expression of *PDCD6* in the DEN group was 2.06 times higher than that of the control group (p<0.05). In the DEN-M group, the expression of *PDCD6* was 1.53 times higher than that of the control group and had a significant difference with the DEN group (p<0.05). However, there was no significant difference in the expression of *PDCD6* among different groups at the end of the sixth month (Figure 3).

**Figure 4:**
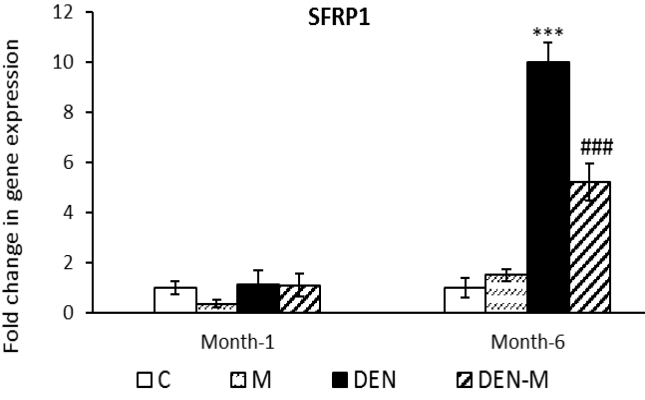
Effect of menthol on mRNA expression of SFRP1 in the liver of mice that received DEN. Results are shown as means ± SEM. Each group contained 5 mice. ***P < 0.001 vs. the control group and ###P < 0.001 vs. the group DEN.

There was no significant difference in the amount of *SFRP1* expression among the groups after the first month. However, by the end of the sixth month, the DEN group showed a 10.00-fold increase in *SFRP1* expression compared to the control group (p<0.001). The DEN-M group showed a 5.23-fold increase in *SFRP1* expression, which was significantly lower than the DEN group (p<0.001) (Figure 4).

**Figure 5:**
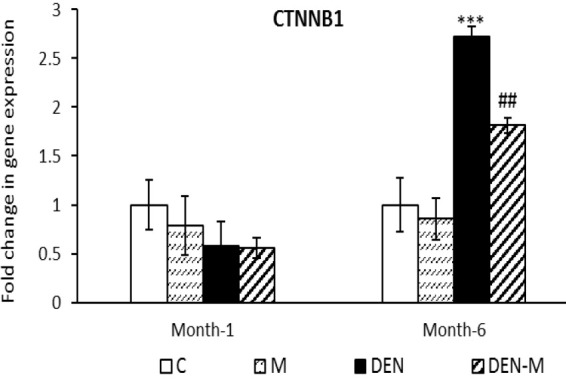
Effect of menthol on mRNA expression of CTTNB1 in the liver of mice that received DEN. Results are shown as means ± SEM. Each group contained 5 mice. ***p<0.001 vs. the control group and ##p<0.01 vs. the group DEN.

At the end of the first month, there was no notable difference in the expression of *CTNNB1* among the different groups. The DEN group showed a 2.72 times increase in its expression compared to the control group (p<0.001) in the second sampling. The DEN-M group, on the other hand, had a significantly lower expression of *CTNNB1* compared to the DEN group (p<0.01), with a 1.81 times increase in expression (Figure 5). 

**Figure 6:**
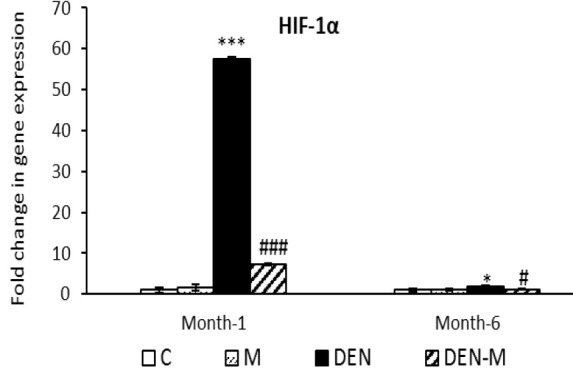
Effect of menthol on mRNA expression of HIF-1α in the liver of mice that received DEN. Results are shown as means ± SEM. Each group contained 5 mice. *p<0.05 and ***p<0.001 vs. the control group and #p<0.05 and ###p<0.001 vs. the group DEN.

In the DEN group, *HIF-1α* expression increased by 57.48-fold at the end of the first month and by 1.8-fold at the end of the sixth month, compared to the control group (p<0.001 and p<0.05, respectively). However, menthol treatment in animals receiving DEN resulted in a significant decrease of *HIF-1α* expression, with a 7.32-fold change at the end of the first month and 1.22-fold at the end of the sixth month (p<0.001 and p<0.05, respectively), as shown in Figure 6. No significant difference in *HIF-1α* expression was observed in the M group compared to the control in either sampling.

**Figure 7:**
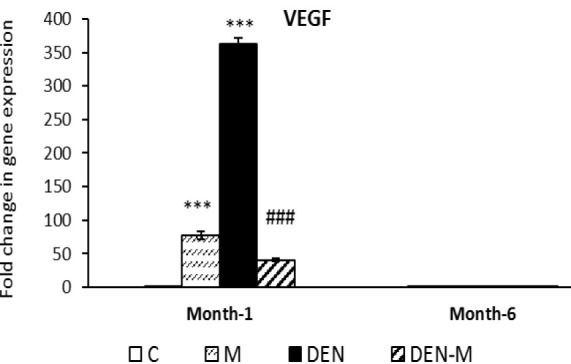
Effect of menthol on mRNA expression of VEGF in the liver of mice that received DEN. Results are shown as means ± SEM. Each group contained 5 mice. ***p<0.001 vs. the control group and ###p<0.001 vs. the group DEN.

In comparison to the control group, the M and DEN groups showed a significant increase in *VEGF* expression by 77.17 and 362.03 times, respectively (p<0.001). The DEN-M group had a lower expression of this growth factor during the first sampling, which was 41.06 times that of the control and significantly lower than the DEN group (p<0.001). In the second sampling, there were no significant differences in *VEGF* expression among the various groups (Figure 7).

### The effect of menthol on histopathological changes in the liver of DEN-treated mice

**Figure 8:**
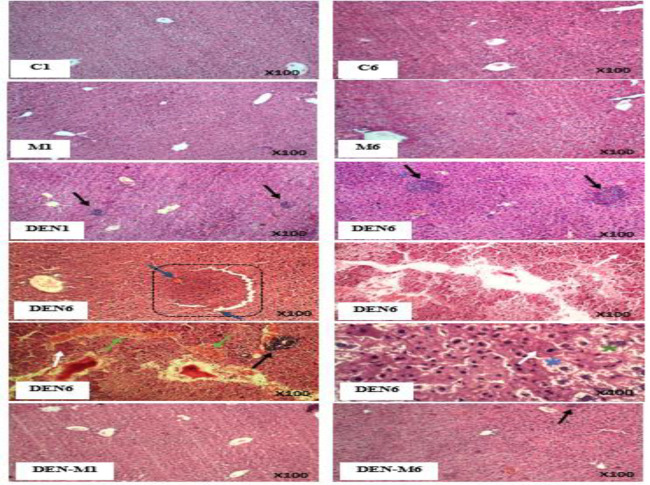
Effect of menthol on the histopathological changes induced by DEN in the liver of mice. The injection of DEN led to the accumulation of inflammatory cells in the liver tissue, as shown by the black arrow in the slides taken during the first and sixth months. This resulted in the disruption of the liver's normal lobular structure, which can be seen in the various slides taken at the six-month mark. Additionally, there was increased blood flow (indicated by the green arrow), hyperplastic nodules (square), and vascular invasion in tumor mass (indicated by the blue arrow). Eosinophilic polygonal cells (indicated by the white arrow), Mallory bodies (indicated by the blue star), and binucleate cells (indicated by the green star) also appeared in the liver after six months. However, treatment with menthol prevented all of these changes in both samplings. C1; control group in month 1, C6; control group in month 6, M1; Menthol group in month 1, DEN1; Diethylnitrosamine group in month 1, DEN6; Diethylnitrosamine group in month 6, DEN-M1; DEN-Menthol group in month 1, DEN-M6; DEN-Menthol group in month 6.

The results of the microscopic examination of the liver tissue showed that intraperitoneal injection of DEN after one month led to the infiltration of inflammatory cells in the liver parenchyma of mice (++), while menthol prevented this phenomenon to a large extent and reduced lobular inflammation in the liver tissue of DEN-M group animals (+). Infiltration of inflammatory mononuclear cells was not observed in the liver parenchyma of the control and M groups. At the end of the sixth month, the intensity of inflammation in the DEN group increased (+++) and the volume of inflammatory cell masses increased. At the end of the sixth month, as in the first month, menthol was successful in reducing the tissue destruction caused by DEN (inflammation intensity: +). In addition, tumor masses with extensive blood flow were observed in the liver tissue of the DEN group. The presence of eosinophilic cells, binucleated cells, and Mallory bodies were also among the characteristics of the liver tissue of DEN group animals in the second sampling. Menthol largely prevented the occurrence of these phenomena in rats receiving DEN (Figure 8). The infiltration of inflammatory cells in the liver tissue of group M was observed at the end of the sixth month (-/+). 

## Discussion

In this study, male mice at 14 days old were injected with DEN. After one month, there was an increase in the expression of *AFP*, *PDCD6*, *HIF-1α*, and *VEGF* in the liver of animals. After six months, there was an increase in the expression of *AFP*, *HIF-1α*, *CTNNB1*, and *SFRP1*. Inflammatory cells were seen in the liver parenchyma at the end of the first month, and severe tissue damage including tumor mass, was observed at the end of the sixth month. These findings align with the results of previous studies (Anwar et al., 2021; Sakai et al., 2022) and indicate that the injection of DEN led to the development and growth of hepatocellular carcinoma in mice.

In light of increasing evidence pointing to the potential therapeutic benefits of various terpenoids, specifically menthol in cancer (Kamran et al., 2022), our research focused on examining the impact of menthol on DEN-induced hepatocellular carcinoma. The dosage of menthol administered in the study was based on its protective effects in the forestomach carcinoma model in mice, as documented by Santo et al. (2021).

In mice injected with DEN, menthol prevented an increase in *AFP* expression in both samplings. According to Zheng et al. (2020), AFP hinders the process of apoptosis in cancer cells by suppressing caspase-3, which ultimately leads to the growth of cancer. It is a widely accepted marker for detecting and tracking the progression of liver cancer. Its overexpression has been linked to cell proliferation, cell motility, and invasive behavior of HCC cells. AFP levels also increase in other liver diseases like hepatitis C, non-alcoholic fatty liver, and cirrhosis (Galle et al., 2019; Hanif et al., 2022). The reduction in *AFP* expression due to menthol in both samples suggests that this compound is highly effective in protecting the liver.

The real-time PCR results have shown that menthol effectively inhibits the increase of *PDCD6* expression during the first sampling. *PDCD6* expression is known to increase in various tumor tissues like the lung, colon, breast, and ovary. By inhibiting apoptosis, it sustains cellular viability in different tumors, thereby preserving their growth (Hashemi et al., 2018). However, recent findings suggest that PDCD6 also contributes to HCC progression through the AKT/GSK3β/β-catenin signaling pathway (Wen et al., 2023). Hence, menthol's ability to decrease *PDCD6* expression by the end of the first month, is indicative of its potential to prevent liver cancer formation and progression in DEN-exposed mice. At the end of the sixth month, the expression of *PDCD6* did not show any significant differences in various groups. Thus, the increased expression of *PDCD6* in hepatocellular carcinoma may be limited to the early stages of the disease.

Recent research by Zheng et al. (2020) indicates that an increase in *AFP* expression leads to an increase in *HIF-1α* expression. Our study confirms this finding, as we observed enhanced expression of *HIF-1α* in both samplings, with particularly high expression at the end of the first month. A meta-analysis by Zheng et al. (2013) identified very high HIF-1α expression as a prognostic factor for HCC development and invasion. Solid tumors are often characterized by hypoxia, which leads to an increase in *HIF-1α *expression and the stimulation of angiogenesis-related gene expression, such as *VEGF* (Wu et al., 2007). Our research showed a 362.03-fold increase in *VEGF* expression in the liver of DEN-treated mice at the end of the first month. However, *VEGF* expression in the same group at the end of the sixth month was not significantly different from the control group. We suggest that HIF-1α/VEGF signaling after the first sampling resulted in widespread angiogenesis in the tumor and damaged the liver. Menthol inhibited hypoxia-induced angiogenesis in the livers of DEN-treated mice by suppressing the expression of both genes, as evidenced by the microscopic appearance of tissue sections from the DEN-M group. Interestingly, *HIF-1α* expression in the M group increased at the first sampling, consistent with findings by Zapata-Guerra et al. (2020), who observed increased *HIF-1α* expression in the liver of *Piaractus brachypomus* under menthol anesthesia.

The CTNNB1 pathway, a key component of Wnt signaling, plays a significant role in HCC. Research has shown that CTNNB1 expression is much higher in the third stage of the disease, which is characterized by vascular invasion, compared to earlier stages (Liu et al., 2016; Fernandes-Ferreira et al., 2022). Our findings also support this trend, as we observed an increase in *CTNNB1* expression in DEN-injected mice during the second sampling. SFRP1, an antagonist of CTNNB1, is typically considered a tumor suppressor gene (Baharudin et al., 2020). However, we found that *SFRP1* mRNA levels were significantly increased in the livers of DEN group mice during the second sampling, which contradicts previous assumptions. Ding et al. (2015) also discovered that SFRP1 is not hypermethylated in HCC and that Wnt signaling overactivation is influenced by other factors beyond SFRP1. While some studies suggest that SFRP1 is a tumor suppressor, recent research has shown that it may have oncogenic properties and could be a useful cancer biomarker (Baharudin et al., 2020). Therefore, the increase in *SFRP1* expression we observed at the end of the sixth month, may contribute to the development and progression of the tumor along with other tumor genes. In DEN-treated mice, the use of menthol treatment prevented an increase in the expression of *CTNNB1* and *SFRP1*. This confirms the antitumor effect of menthol.

The liver tissue of mice injected with DEN was examined microscopically, revealing that inflammatory cells had infiltrated the liver parenchyma. Over six months, the number and volume of inflammatory masses increased. Lobular infiltration was observed as a result of nitrosamine treatment and was linked to elevated expression of inflammatory factors (Fu et al., 2020; Wang et al., 2023). Treatment with menthol significantly reduced liver inflammation in the DEN-M group. Research has shown that menthol has anti-inflammatory properties (Rozza et al., 2014; Du et al., 2020), hence it is recommended to assess the expression of pro-inflammatory cytokines in different study groups. In the second sampling, DEN injection caused extensive tissue damage, such as disruption of lobular structure, the presence of eosinophil cells and Mallory bodies, vascular expansion, and increased tissue blood flow around tumor masses. These histopathological changes are consistent with the findings of previous studies (Santos et al., 2014, Santos et al., 2017). Menthol prevented the development of these changes in the liver tissue, as predicted by molecular investigations. Based on the findings of mild lobular inflammation in the liver of animals in group M after six months of treatment, it appears that prolonged use of menthol could have negative effects.

Overall, our research indicates that menthol can protect the liver from DEN-induced carcinoma by inhibiting Wnt signaling and angiogenesis and preventing histopathological changes. These findings provide evidence that menthol possesses anti-liver cancer properties.

**Table1:** Primer sequences used in real-time PCR

**Sequences**		**Gene**
5'-GTTGTCTCCTGCGACTTCA-3'	F	*GAPDH*
5'-GGTGGTCCAGGGTTTCTTA-3'	R	
5'-TTGTGTATAAGGAATGAAGCAAG-3'	F	*AFP*
5'-CCTGTTGGAATACGAAGAGTT-3'	R	
5'-ATCCAAAGAGTAGCTGCAGG-3'	F	*CTNNB1*
5'-TCATCCTGGCGATATCCAAG-3'	R	
5'-GCAAGCGAGTTTGCACTGAG-3'	F	*SFRP1*
5'-CCGCTTCAGCTCCTTCTTCT-3'	R	
5'-AGAAGTTTGGGGAAGAGATCG-3'	F	*PDCD6*
5'-CGAGAGGCCACATTCTTGAT-3'	R	
5'-AAAGGCTTCAGTGTGGTCTGAGAG-3'	F	*VEGF*
5'-GGTTGGAACCGGCATCTTTATC-3'	R	
5'-TGCTTGGTGCTGATTTGTGA-3'	F	*HIF-1α*
5'-GGTCAGATGATCAGAGTCCA-3'	R
